# Supplementary material for: Serum Levels of Urokinase Plasminogen Activator Receptor (suPAR) Discriminate Moderate Uncontrolled from Severe Asthma
Source: J Pers Med. 2022 Oct 28;12(11):1776. doi: 10.3390/jpm12111776 (PMC9693242; doi:10.3390/jpm12111776)
Supplement: Supplementary file 1 [file jpm-12-01776-s001.zip › jpm-1954642-supplementary.pdf]

Supplementary Table S1. Correlation analysis between suPAR levels and age, BMI, T2 biomarkers, blood cell count, CRP, electrolytes, and parameters of the kidney and liver function

		Age (years)	FeNO (ppb)	FEV1 / FVC	Eosinophils (%)	Absolut eosinophil count (cells /μL)	White blood cell count (cells /μL)	CRP (mg/dL)	Urea (mg/dL)	Cr (mg/dL)	AST (mg/dL)	ALT (mg/dL)	γGT (mg/dL)	K (mEq/L)	Na(mEq/L)	BMI (kg/m2)
suPAR (ng/mL)	Pearson Correlation	0.140	-0.144	0.020	-0.037	-0.016	-0.576	0.050	0.131	-0.610	-0.086	-0.091	-0.070	-0.020	-0.028	0.079
	Sig. (2-tailed)	0.238	0.309	0.870	0.767	0.895	0.424	0.688	0.700	0.582	0.751	0.738	0.574	0.890	0.908	0.646

Abbreviations: ALT, alanine transaminase; AST, aspartate transaminase; BMI, body mass index; Cr, creatinine; CRP, c-reactive protein; FeNO, fractional exhaled nitric oxide; FeV1/FVC, forced expiratory volume in the first second / force vital capacity; γGT, gamma-Glutamyl Transpeptidase; K, potassium; Na, sodium
